# Array comparative hybridisation reveals a high degree of similarity between UK and European clinical isolates of hypervirulent *Clostridium difficile*

**DOI:** 10.1186/1471-2164-11-389

**Published:** 2010-06-21

**Authors:** Gemma L Marsden, Ian J Davis, Victoria J Wright, Mohammed Sebaihia, Ed J Kuijper, Nigel P Minton

**Affiliations:** 1 Centre for Biomolecular Sciences, School of Molecular Medical Sciences, Nottingham Digestive Diseases Centre NIHR Biomedical Research, University of Nottingham, Nottingham, NG7 2RD, UK; 2 Wellcome Trust Genome Campus, Hinxton, Cambridge, CB10 1SA, UK; 3 Department of Medical Microbiology, Centre for Infectious Diseases, Leiden University Medical Centre, Leiden, The Netherlands; 4 The School of Pharmacy University of London, 29-39 Brunswick Square, London WC1N 1AX, UK

## Abstract

**Background:**

*Clostridium difficile *is a Gram-positive, anaerobic, spore-forming bacterium that is responsible for *C. difficile *associated disease in humans and is currently the most common cause of nosocomial diarrhoea in the western world. This current status has been linked to the emergence of a highly virulent PCR-ribotype 027 strain. The aim of this work was to identify regions of sequence divergence that may be used as genetic markers of hypervirulent PCR-ribotype 027 strains and markers of the sequenced strain, CD630 by array comparative hybridisation.

**Results:**

In this study, we examined 94 clinical strains of the most common PCR-ribotypes isolated in mainland Europe and the UK by array comparative genomic hybridisation. Our array was comprehensive with 40,097 oligonucleotides covering the *C. difficile *630 genome and revealed a core genome for all the strains of 32%. The array also covered genes unique to two PCR-ribotype 027 strains, relative to *C. difficile *630 which were represented by 681 probes. All of these genes were also found in the commonly occuring PCR-ribotypes 001 and 106, and the emerging hypervirulent PCR-ribotype 078 strains, indicating that these are markers for all highly virulent strains.

**Conclusions:**

We have fulfilled the aims of this study by identifying markers for CD630 and markers associated with hypervirulence, albeit genes that are not just indicative of PCR-ribotype 027 strains. We have also extended this study and have defined a more stringent core gene set compared to those previously published due to the comprehensive array coverage. Further to this we have defined a list of genes absent from non-toxinogenic strains and defined the nature of the specific toxin deletion in the strain CD37.

## Background

*Clostridium difficile (C. difficile) *is a Gram-positive, spore-forming, anaerobic bacterium currently responsible for virtually all cases of pseudomembranous colitis (PMC) and for 10-25% of cases of antibiotic-associated diarrhoea [1]. The organism is resistant to various antibiotics and capitalizes on the ensuing disruption of the normal intestinal flora to colonization and cause disease. The spectrum of disease ranges from asymptomatic carriage to a fulminant, relapsing, and increasingly fatal colitis [2]. The effects of *C. difficile *infection (CDI) are devastating, both in terms of morbidity and mortality and the high costs of disease management [3,4]. Once regarded as relatively uncommon, there has been an upward trend in the incidence of CDI in both North America [1,5,6] and Europe [7,8] culminating in 2007 in over 5 times as many deaths (8,324) than MRSA (1,593) in England and Wales [9].

Various reasons have been suggested for this extraordinary rise in incidence and mortality, including the emergence of so-called 'hypervirulent' strains. The most prominent such strains belong to PCR-ribotype 027, responsible in North America for a 5-fold increase in the historical average of CDI, more severe disease, higher relapse rates, increased mortality, and greater resistance to fluoroquinolone antibiotics [10]. Although restriction endonuclease analysis (REA) and multilocus variable number tandem repeat analysis (MLVA) have greater powers of discrimination [11], PCR-ribotyping [12,13], represents the most widely used method of distinguishing strains, and relies on the use of specific primers complementary to the 3' end of the 16S rRNA gene and to the 5' end of the 23S rRNA gene to amplify the variable-length intergenic spacer region. The fragments generated are analysed electrophoretically, and the size distribution of fragments obtained compared to reference patterns. Presently upwards of 150 PCR-ribotypes are recognised [14].

Typically, PCR-ribotype 027 strains (also characterised as toxinotype III, North American pulsed field gel electrophoresis type 1, NAP1, and restriction endonuclease analysis group BI) possess a binary toxin gene and encode a variant TcdC repressor protein suggested to account for increased toxin production [15,16]. Current PCR-ribotype 027 strains have, since the first documented isolate [17], acquired resistance to fluoroquinolone and erythromycin antibiotics [18-20], and their occurrence is often associated with an excessive use of quinolone antibiotics. The speed with which PCR-ribotype 027 can become predominant is exemplified by events in the UK where its incidence increased from virtually zero over the period 1990 to 2005 [21], to 25.9% through the period 2005 to 2007 [22] to 41.4% across England over the period April 2007 to March 2008 [23]. However, whilst PCR-ribotype 027 strains have received much attention, other strains may also present an equivalent threat in terms of disease severity. In many countries, different PCR-ribotypes can predominate, but be extremely rare elsewhere. For instance, the PCR-ribotype 106, although common in the UK [22], was entirely absent from the European study of Barbut *et al*. [24]. In the Netherlands, PCR-ribotype 078 increased from 3% to 13% over the period February 2005 to February 2008, infected younger individuals than PCR-ribotype 027 and was more frequently involved in community-associated disease [25]. Human PCR-ribotype 078 isolates possess a number of features in common with PCR-ribotype 027 and have recently been shown to be genetically related to isolates from pigs [26].

Currently, the overall reason why particular strains achieve epidemic status is unclear. Although some suggestions have been made [27], in terms of altered toxin production, presence of binary toxin, changes in antibiotic susceptibility and sporeogenesis, the situation is likely to be more complex involving a number of different phenotypic traits. A previous comparative phylogenomic study using microarrays based only on those genes present in the annotated genome sequence of a PCR-ribotype 012 strain, CD630 [28,29] [GenBank: AM180355.1]), showed that the PCR-ribotype 027 strains tested formed a tight clade, which was distinct from the other 56 strains analyzed and confirmed the clonal nature of PCR-ribotype 027 strains, but indicated extensive variation in the genetic content [29]. A further study microarray study included extra genes from the Canadian PCR-ribotype 027 strain, QCD-32g58 [30] [Genbank: AAML00000000]) where the conserved genetic core was defined and divergent regions were conserved amongst strains of the same host origin.

The aim of this current study was to identify unique strain differences using a genome wide approach, with a view to both gaining greater insight into enhanced virulence and as a means of identifying regions of sequence divergence suitable for use as diagnostic indicators of hypervirulence. To accomplish this, a DNA microarray comprised of over 41000 oligonucleotides was designed and constructed using *in situ *inkjet oligonucleotide synthesis. The strains represented included CD630, R20291 and QCD-32g58. The strains subjected to comparative genomic hybridisation were chosen as they represented the most prevalent PCR-ribotypes from the UK and EU [2,22]. The work presented in this study represents the application of a novel microarray format to the study of comparative genomic hybridisation and is the only study that employs the widely used molecular typing technique of ribotyping to choose the strains for hybridisation and for subsequent clustering analysis.

## Results

### Array verification and coverage

Forty thousand and ninety seven 60-mer probes were designed to cover the sequenced and annotated genome of C. *difficile *CD630. This essentially corresponded to a probe every approximately 200 bp. A further 687 probes were designed to extra genes in the preliminary 454 sequence produced for R20291 by the Sanger in 2007 and the available unannotated QCD-32g58 sequence. Additionally, 17 extra genes including the toxin genes, *cwp66 *and *slpA *were represented at high density by 346 oligonucleotides. Initial experiments were performed with a set of control strains that included CD630, R20291, R23052 and CD196 (R12087). A CD630 self-self hybridisation was also performed. Analysis of the data obtained showed that the genome of strain CD630 hybridised to 57 of the '027-specific' probes; and a BLASTN *in silico *analysis of the array oligonucleotides against the CD630, R20291 and QCD-32g58 sequences used to design the array showed that these oligonucleotides had highly significant matches in these strains. Accordingly, these oligonucleotides were excluded from further analysis.

**Figure 1:**
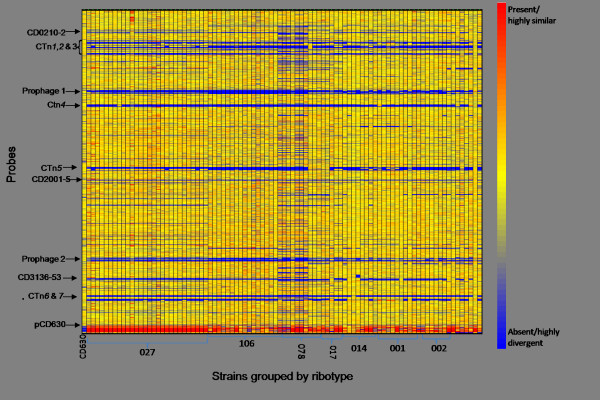
**Comparative genomic analysis of 94 strains of clinical strains of *C. difficile***. The probes were arranged by their corresponding *C. difficile *630 gene, with CD0001 at the top and CD3680 at the bottom, followed by CDS from the plasmid pCD630 (CDP01 to CDP11) and finally probes representing the genes unique to ribotype 027. Each column represents an isolate, and each row corresponds to a probe. The status of each probe is indicated by color as follows: red, present/conserved in the test strain; blue, absent in the test strain and yellow present in both the test and control strains. The strains are grouped by PCR-ribotype and this is indicated below. The writing on the left indicates regions of divergence from CD630 in all of the strains tested.

Analysis of the remaining PCR-ribotype 027 oligonucleotides with genomic DNA of the control strains showed that these probes produced a positive signal with the DNA of PCR-ribotype 027 strains. Figure 1 shows a condition tree clustering for all the strains against all of the probes, with those representing CD0001 listed at the top, pCD630, the extra genes and finally the extra PCR-ribotype 027 genes at the bottom. Strains are grouped by PCR-ribotype and, on initial inspection, demonstrate that each PCR-ribotype exhibits a visually similar pattern of hybridisation. Additional files 1, 2, 3, 4, 5, 6, 7 and 8 presents a full list of the probes that are present or absent in each strain.

### Core

The core gene list was established by examining CD630 probes present and at a 1:1 ratio for each strain. Analysis of the core genes for all the strains tested showed that 32% of CD630 probes were conserved (12788/40097). This percentage is higher than those previously published in other array studies of 19.7% [29] and 16% [30]. This is perhaps surprising due to the wide variety of PCR-ribotypes analysed but as this array is denser, containing more than one reporter element per gene, greater sequence conservation will be evident than for arrays with one reporter per gene. Therefore, genes such as *slpA *which may not be included in the previously reported core percentages would be represented in this current figure. Our array also covers intergenic regions not covered by lower density microarrays. Conservation of genes was seen amongst all functional categories (see Additional file 9). Even greater conservation was seen when comparing strains of the same PCR-ribotype and Table 1 indicates the percentage conservation amongst the studied PCR-ribotypes, with a conservation of 85% or more for PCR-ribotypes 003, 012, 014 and 020.

**Table 1:** Percentage core conservation by PCR-ribotype (by probe)

PCR-ribotype	CD630 Probes present	Percentage conservation (%)
001	30127	75.1

002	28559	71.2

003*	34451	86.1

012*	39777	99.2

014	30482	76.0

015*	35122	87.6

017	28012	69.9

020*	36165	90.2

027	17224	43.0

078	19318	48.2

106	16451	41.0

Total	12789/40097	31.8

*indicates only one reference strain tested.

### Mobile elements

*C. difficile *is known to have a highly mosaic genome with many mobile genetic elements such as conjugative transposons and prophages [28]. Of the 1392 probes representing mobile or extrachromosomal elements in strain CD630, only 92 probes were present in the core of all the strains hybridised. Additional file 10 summarises the presence of the known CD630 mobile elements in each PCR-ribotype. In the majority of PCR-ribotype 027 and 001 strains, CTn*1 *is absent or highly divergent. It is absent or highly divergent in all PCR-ribotype 078 and 015 strains. CTn*2 *is absent from all of the PCR-ribotypes except from the PCR-ribotype 12 strains, CD630 and ECDC 012. CTn*3 *is absent or highly divergent from all PCR-ribotypes except 078 and 012. CTn*3 *or Tn*5397 *is the only known mobile *C. difficile *element containing erythromycin and tetracycline resistance [31]. Therefore resistance to these antibiotic classes in any of the strains tested, including R20291 and PCR-ribotypes 078 and 106 strains (which are resistant to erythromycin) must be provided by an as yet undefined genetic element or mutation [32,33].

CTn*4 *is detectable in the PCR-ribotype 027 Quebec strain 23M63 but is absent or highly divergent in 27/28 of the PCR-ribotype 027 strains tested on the array utilised in this study. It is also partially present in one of the PCR-ribotype 001 strains tested but absent from all the other PCR-ribotypes. CTn*5 *is absent or highly divergent in 6 PCR-ribotypes; 001, 002, 014, 015 003 and 020. In all PCR-ribotype 017 strains only genes CD1864-9 are absent or highly divergent. These genes are also absent or highly divergent in 1-2 strains of the remaining 3 PCR-ribotypes; 027, 078 and 106. One PCR-ribotype 014 strain exhibits hybridisation between CD3330-44, but CTn*6 *is absent or highly divergent in all the other strains tested. Conversely Ctn*7 *is present in some form in all PCR-ribotypes except PCR-ribotypes 002 and 015. Prophage 1 is absent from all the strains tested except the PCR-ribotype 012 strains. Prophage 2 hybridises between CD2927-59 in all but PCR-ribotype 001, 002, 014, 015 and 078 strains.

## Virulence genes

Various genetic loci that have been implicated in the virulence and pathogenesis of *C. difficile*, including those encoding for toxins and putative adhesions, as well as factors responsible for the spread of *C. difficile*, such as flagella and motility genes, antibiotic resistance and regulatory genes.

### Toxins

The *C. difficile *genome contains the PaLoc (pathogenicity locus) which harbours five genes (*tcdABCDE*) responsible for the synthesis and regulation of the two major virulence factors, toxins, TcdA and TcdB. Variation in this region is extensive and as a consequence toxinotyping is a frequently used molecular method used to discriminate between strains [34,35]. Variable sequences include both the structural genes encoding the toxins, and the associated regulatory genes. Thus, the ability of some PCR-ribotype 027 strains to produce more of both toxins is attributed to a deletion at position 117 in the negative regulator of toxin production, *tcdC *[15,16], leading to a truncated TcdC protein. The occurrence of similar deletions in other strains not generally associated with epidemics suggests, however, that such changes are not indicative of hypervirulence [20]. PCR-ribotype 027 strains are usually toxinotype III strains, whereas CD630 is toxinotype 0.

The array results confirm that *tcdB *is conserved among all PCR-ribotype 027 isolates examined and diverged in the 3' region of *tcdC *(the negative regulator of toxin production) as indicated by a lack of hybridisation to EXP_CD630_800001_805000_s_PSO-60-77, the last *tcdC *probe on the array. Naturally occurring toxin A-B+ strains cause diarrhoea and colitis in humans [36] and are generally PCR-ribotype 017 (toxinotype VIII). From the observed hybridisation obtained with our array, all of the PCR-ribotype 017 strains examined here lacked *tcdA *and exhibited divergence in *tcdB *when compared to the corresponding CD630 and SM probes (data not shown).

Some *C. difficile *strains also produce a third toxin in addition to TcdA and TcdB, a binary ADP-ribosyltransferase toxin encoded by *cdtA *and *cdtB*. The role of binary toxin in pathogenesis is unclear, although it has been linked to increased disease severity [2]. The genes *cdtA *and *cdtB *are conserved in PCR-ribotypes 027 and 078. Our hybridisation results agree with those previously reported for CD630, showing divergence in both of these genes which cause these genes not to be active in this strain [37]. PCR-ribotype 017 also displays similar results to previous publications and, shows limited hybridisation to some CD630 *cdtA *and *cdtB *reporters as concluded by Rupnik [35]. The results from this study for the other PCR-ribotypes examined show that this region is divergent.

### Flagella and motility genes

Flagella are important in pathogenesis for many enteric pathogens including *Campylobacter jejuni *and *Salmonella enterica *serovar Enteridis [38,39]. Chemotaxis and motility are inextricably linked and both are important for bacterial survival allowing the bacteria to move towards nutrients and away from substances that may prove detrimental.

Genes that allow for flagella modification by glycosylation have recently been described in *C. difficile *QCD-32g58 and R20291 upstream of the flagellar biosynthesis locus [32,40]. Reporters representing 2 of the 4 loci (CDR0223 and 5) are present on the array and are conserved in all strains but two PCR-ribotype 017 strains (L22 and 23). Stabler *et al *[32] described the flagella related genes in 2 loci of the CD630 genome, F1; CD0226-40 and F3; CD0245-71 [29]. Loss of, or significant divergence in the F1 and inter-flagella region (F2; CD0241-4) was observed in PCR-ribotype 027 strains; this was shown to be due to 84-90% sequence identity in this region [32].

Our data shows that only 7/93 strains are divergent in these genes and this includes the two PCR-ribotype 017 strains discussed above, two non-toxigenic strains and two PCR-ribotype 078 strains. PCR-ribotype 078 strains have previously been reported to be non-motile [32] and although the CD630 flagella loci appears to be highly divergent or absent in these stains, the corresponding R20291 flagella and flagella glycosylation genes are present, indicating that another mechanism of variation is responsible for their non-motility.

### Antibiotic resistance

Another contributing factor to the spread of *C. difficile *infection is the acquisition of antibiotic resistance. The genome sequence of CD630 allowed the identification of many genes associated with antibiotic resistance, including those already known such as *ermB *and *tetM*, and those with no prior experimental data, such as the putative lantibiotic antibiotic resistance genes (CD0478-CD0482, CD0820-CD0824 and CD1349-CD1352). In contrast to strain CD630, the epidemic 027 strains have been shown to be highly resistant to fluoroquinolones due to point mutations in the DNA gyrase genes which cannot be detected by this microarray [4,29].

In agreement with previous array data, the lantibiotic resistance loci, CD0643-6 and CD01349-52 are absent or highly divergent in all the PCR-ribotype 078 strains tested and appear to be divergent in some of the tested PCR-ribotype 027 strains. The putative ABC transporter that confers daunorubicin resistance (CD0456) was absent from PCR-ribotype 078, 106 and 020 strains, but present in all others. The R20291 sequence showed that chloramphenicol resistance was conferred by CDR3461, part of the CTn*027*. The array shows that this gene or its homologue is present in all of our PCR-ribotype 027 and 001 strains, present in the majority of PCR-ribotype 078 strains and divergent in the remaining PCR-ribotype strains.

### Regulatory systems

Regulatory genes form a large part of the *C. difficile *genome comprising 11% of the CD630 genome [28]. In *Staphylococcus aureus*, the *agr *quorum sensing locus (*agrCABD*) has been implicated as a key regulator of many virulence factors [41,42]. In strain CD630 only homologues of *agrD *and *agrB *were present, respectively encoding a prepeptide of a secreted small autoinducer peptide and a transmembrane protein involved in AgrD processing. The homologous system in *S. aureus *also contains two further genes; *agrC *and *agrA *encoding a two-coponent system. Preliminary 454 sequencing of the PCR-ribotype 027 had shown that R20291 contained a second complete copy of an *agr *locus (*agrCABD*) in addition to the *agrBD *genes of strain CD630. Accordingly, oligonucleotides corresponding to this extra *agrCABD *locus was incorporated on our array at high density with an additional 25 probes.

**Figure 2:**
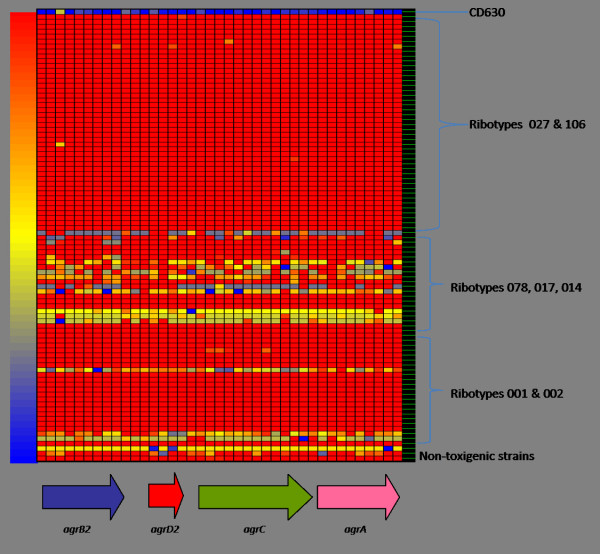
**Schematic diagram produced by GeneSpring showing all the oligonucleotides representing the second *agr *locus against all the clinical strains**. The gene context of region is detailed below the diagram but this is not to scale. Each row represents an isolate, and each column corresponds to a probe. Strain PCR-ribotypes are indicated on the right. The status of each probe is indicated by color as follows: red, present/conserved in the test strain; blue, absent in the test strain and yellow in this case were the region is absent in CD630 indicates divergence in these genes.

Hybridisation against our array demonstrated that the extra *agrCABD *locus found in R20291 is entirely present in the genomes of 82 of the 94 (86%) strains tested, including two of four non-toxigenic strains (Figure 2). Additional file 11 details the presence, absence and divergence (signal around 1) for each probe in the remaining 12 strains. The hybridisation to a few probes by DNA isolated from each of these 12 strain implies that this region is divergent rather than absent. PCR primer walking was performed on the strains detailed in Additional file 12 and primers were designed to the region CDR3184-3190. These primers generated amplicons of the expected size when DNA was derived from the positive control, R20291. No such amplicons were generated when DNA was derived from the 12 test strains. The positive control primers designed to amplify CDR3190 produced an amplicon with DNA isolated from all strains (data not shown). Overall, these results indicate that the absence of this additional *agrCABD *locus is the exception, rather than the rule.

### Other virulence factors

The ability to sporulate is an important mechanism for the dissemination of all clostridia. A recent study has suggested that epidemic PCR-ribotype 027 isolates are more prolific in terms of spore formation than non-epidemic strains [43]. The sporulation related genes represented on the array are conserved throughout all the strains tested.

Another set of genes that have been implicated in virulence are those encoding cell surface proteins, including Cwp84 [44]. The majority of the genes coding for cell surface proteins are conserved in all of the strains tested. The genes which appear to show divergence are *cwp66*, CD2791 and CD3392.

## Non toxigenic strains

**Figure 3:**
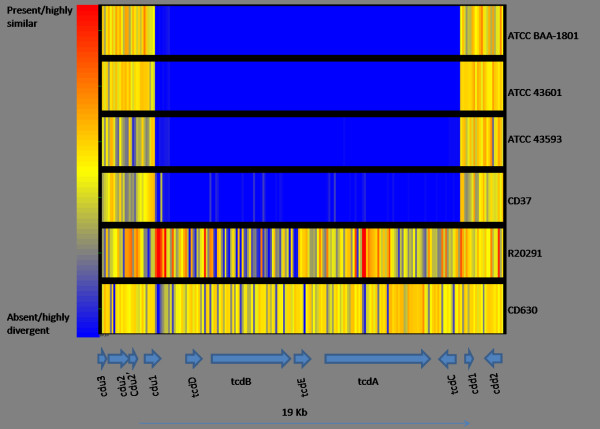
**Schematic diagram produced by GeneSpring showing all the oligonucleotides representing the PaLoc against CD630, R20291 and the 4 non-toxigenic strains tested**. This includes probes that will not hybridise to either of our control strains, CD630 and R20291. The gene context of the region is detailed below the diagram but this is not to scale. Each row represents an isolate, and each column corresponds to a probe. The strain ID is indicated on the right. The status of each probe is indicated by color as follows: red, present/conserved in the test strain; blue, absent in the test strain and yellow present in both the test and control strains.

In order to provide further validation of the array, the DNA of a total of four non-toxigenic strains (CD37, ATCC 43593 (1351), ATCC BAA-1801 (3232) and ATCC 43501 (7322)), were hybridised to the array. Braun *et al *[45] defined the integration site for the pathogenicity locus (PaLoc) by the sequence-based comparison of toxigenic strains and non-toxinogenic strains. Included in this analysis were the three ATCC non-toxigenic strains 43593, 43501 and BAA-1801. The *C. difficile *strain CD37 has previously been described as non-toxigenic but the nature of the deletion never fully characterised [46]. As shown in Figure 3, the PaLoc is absent from all four non-toxigenic strains at the site determined by Braun *et al *[45]. In these strains, the *cdu1 *gene is adjacent to the *cdd1 *gene and this was confirmed using the multiplex PCR and primers described by Braun *et al*., [45] (data not shown).

Further analysis of the non-toxigenic strains was performed and showed that 71 genes were absent or highly divergent from all of these strains compared to CD630 and 15 of the R20291 extra genes were also absent or highly divergent (detailed in Additional file 13). These genes include coding sequences (CDS) in the conjugative transposons CTn*2 *and CTn*6*. Two of the strains have additionally lost, or are highly divergent in, the flagella genes CD0226-40 and the R20291 flagella F2 region CDR0242-7.

## Discussion

The microarray used in the current study was designed to cover one sequenced strain of *C. difficile *(CD630), and the preliminary unannotated sequence from two different PCR-ribotype 027 strains, R20291 (based on a 454 sequence run available at ftp://ftp.sanger.ac.uk/pub/pathogens/cd/C_difficile_Bi_454.dbs) and QCD-32g58. Since the microarray was designed in 2007, the fully annotated sequence of R20291 [EMBL: FN545816], together with the historical PCR-ribotype 027 strain CD196 [EMBL: FN538970], has been published [32]. Comparison of the 027-specific probes on the microarray to the published sequence of R20291 has revealed some differences. In particular, a total of 234 additional R20291 genes were described in comparison to the sequenced strain CD630 and 505 genes were found to be unique to CD630. The array used in our study covers 169 of the 234 additional genes (72.2%). The missing CDS are detailed in Table 2. The majority of genes not represented on the array are transposon or phage related (40 genes) and the remaining 25 genes have oligonucleotide reporters representing neighbouring genes on the array.

**Table 2:** The 027 genes absent from all PCR-ribotypes except 001, 078 and 106

Gene	Function
CDR0043-7	thymidylate synthase, dihydrofolate reductase region, putative uncharacterized protein, thiamine biosynthesis protein thic, putative thymidylate synthase

CDR0310	tetR (putative transcriptional regulator)

CDR0531	putative membrane-associated metalloprotease

CDR0551	abc transporter, atp-binding/permease protein

CDR1277-8	putative uncharacterized protein, transcriptional regulator, araC family protein

CDR1324	putative uncharacterized protein

CDR1416-7	putative uncharacterized protein, hipa-like

CDR1443	phage-related protein

CDR1446	prophage antirepressor-related protein

CDR1448-9	putative uncharacterized protein; putative phage tail tape measure protein

CDR1456-5	putative phage tail fiber protein, hypothetical phage protein

CDR2757-61	putative uncharacterized protein, putative lantibiotic ABC transporter, ATP-binding protein, sortase, two-component system, sensor histidine kinase, putative uncharacterized protein

CDR2961	putative uncharacterized protein

CDR2986-92	putative uncharacterized protein x5, frg domain protein

CDR2994	crispr-associated helicase cas3

CDR2996-9	putative uncharacterized protein, crispr-associated protein cas6, crispr-associated protein cas5 family, putative uncharacterized protein

CDR3010	phage-related protein

CDR3025	gcn5-related n-acetyltransferase;

CDR3278	putative exported protein

CDR3280	putative uncharacterized protein

CDR3281	transposon tn21 resolvase

CDR3285-7	putative uncharacterized proteins

CDR3458-9	putative conjugative transposon replication initiation factor

CDR3462-3	conjugative transposon protein × 2

CDR3466	conjugative transposon protein

During the gap closure sequencing and subsequent analysis of the R20291 and CD196 genomes, 47 extra genes were found in strain R20291 compared to the historical strain CD196. This included a unique 20 Kb phage island, termed SMPI1 which was found to be inserted into a unique PCR-ribotype 027 conjugative transposon, named CTn*027*. Our array was designed prior to gap closure of these 2 genomes and as a consequence represents only 29.8% of the 47 additional genes found in the R20291 genome. The majority of the genes not represented by the array form part of the conjugative transposon, CTn*027*, which is unique to R20291. However, the 14 CTn*027 *genes that are represented by our array were found to be present in the genomes of only 5 of the 28 PCR-riboytpe 027 strains tested, thereby indicating that this transposon is not common amongst PCR-riboytpe 027 strains.

The tiling nature of our array has established a more stringent and definitive core gene or sequence list than those previously published. Analysis of the core genes for all the strains tested showed that 32% of CD630 probes were conserved (12788/40097). This percentage is higher than those previously published in other array studies of 19.7% [32] and 16% [30]. The high density of our array, the fact that there is more than one reporter per gene and the coverage of intergenic regions means that our array provides a greater ability to define the core genes or sequences in each strain than PCR-spotted or single reporter per gene arrays. Conservation of genes was seen amongst all functional categories (Additional file 9).

As expected even greater conservation was seen when comparing strains of the same PCR-ribotype. Table 1 indicates the percentage conservation amongst the studied PCR-ribotypes, with conservation of 85% or more for PCR-ribotypes 003, 012, 014 and 020 in comparison to strain CD630. However, three of these ribotypes were only represented by one isolate and the study would have to be extended to include more isolates to provide a real indication of conservation among ribotypes 003, 012 and 020. Our array confirmed divergence between strains within the toxin encoding regions between PCR-ribotypes, particularly in the case of *tcdB *and *cdtAB*, while at the same time demonstrated that the particular *tcdB *variant present in R20291 was conserved amongst all PCR-ribotype 027 isolates tested.

Examination of the conjugative transposons in different PCR-ribotypes of *C. difficile *shows that the pattern of hybridisation to the probes representing the mobile elements provides only a limited indication of PCR-ribotype. Thus, while the majority of PCR-ribotype 106 strains lack any sequences homologous to CTn*5*, one strain (L25) does carry CTn*5*-derived sequences. Many strains showed homology to the genes at the terminal ends of the transposons. Whilst this could be because these genes are common to many transposons, genes such as CD3325 and CD3349 of CTn*6 *are present in all the strains tested even though the occurrence of the whole tranposon is limited to CD630 and one PCR-ribotype 014 strain. The two single PCR-ribotype 003 and 015, and the eight PCR-ribotype 002 strains appear particularly devoid of homology to the specific transposons and prophages probes present on the array. The elements tested appear completely absent from six of the eight PCR-ribotype 002 strains, as well as the single PCR-ribotype 003 strain (aside from partial hybridisation to some CTn*1 *probes), and PCR-ribotype 015 strain (aside from partial hybridisation to some prophage 2 probes). Two of the PCR-ribotype 002 strains carry some regions with limited homology to parts of prophage 2.

A major aim of the study was to determine whether it was possible to identify divergent sequences that may be characteristic of either PCR-ribotype 027, or indeed hypervirulence. Seventeen of the 537 PCR-ribotype 027 probes represented on the microarray were present in all of the strains (Table 3). *In silico *analysis showed that these matches were not expected against the available non-027 nucleotide sequences. Determination of hypervirulent sequence markers to separate PCR-ribotype 027 strains from the rest of the strains was not possible. All of the 027 genes represented by the 027 probes were present in at least one strain of PCR-ribotypes 001, 020, 078 and 106. Table 4 details the percentage of 027 probes present in each PCR-ribotype. Additional file 14 details the 027 genes discovered by Stabler *et al *[32] absent from the array design. Additional file 15 examines the probes absent in each PCR-ribotype. Filtering was performed to see if any elements on the array could be used to identify individual PCR-ribotypes. No single probe was representative of just one PCR-ribotype.

**Table 3:** PCR-ribotype 027 probes found to be present in all strains

Probe name	Gene	Probe number
EXP_CDxSM0220_1_873_s_PSO-60-0074	CDR0223	40262

EXP_CDxSM0239_1_1878_s_PSO-60-1630	CDR0242	40273

EXP_CDxSM0240_1_2115_s_PSO-60-1758	CDR0243	40276

EXP_CDxSM0243_1_945_s_PSO-60-0764	CDR0246	40287

EXP_CDxSM0444_1_474_s_PSO-60-0048	CDR0427	40323

EXP_CDxSM1786_1_486_s_PSO-60-0366	CDR1847	40468

EXP_CDxSM1787_1_1176_s_PSO-60-0532	CDR1848	40472

EXP_CDxSM1788_1_1458_s_PSO-60-1128	CDR1849	40476

EXP_CDxSM1789_1_1203_s_PSO-60-0160	CDR1850	40477

EXP_CDxSM2443_1_1296_s_PSO-60-1098	CDR2514	40519

EXP_CDxSM2444_1_1443_s_PSO-60-1118	CDR2515	40522

EXP_CDxSM2445_1_1128_s_PSO-60-0954	CDR2516	40525

EXP_CDxSM2446_1_651_s_PSO-60-3	CDR2517	40528

EXP_CDxSM2827_1_2064_s_PSO-60-1272	CDR2908	40545

EXP_CDxSM2828_1_3336_s_PSO-60-2852	CDR2909	40549

EXP_CDxSM2829_1_429_s_PSO-60-19	CDR2910	40551

EXP_CDxSM2833_1_1464_s_PSO-60-1076	CDR2912	40563

**Table 4:** Percentage of PCR-ribotype 027 probes represented on the microarray that are present in each PCR-ribotype

PCR-ribotype	027 Probes present	Percentage conservation (%)
001	537	100.0

002	157	29.2

003*	349	65.0

012*	512	95.3

014	85	15.8

015*	533	99.2

017	212	39.5

020*	537	100.0

027	531	98.8

078	537	100.0

106	537	100.0

*indicates only one reference strain tested

It was noteworthy that the PCR-ribotype 020 reference strain also shares the extra PCR-ribotype 027 genes. PCR-ribotypes 020 and 014 are very difficult to differentiate by PCR-ribotyping and, therefore, frequently combined as "014/020 type". This 014/020 PCR-ribotype is currently the most frequently found type in Europe. It is remarkable, however, that type 014 differed considerably from 020 by the presence of extra 027 genes, indicating that the reference PCR-ribotype strains of 020 and 014 are clearly different. As only one reference strain of PCR-ribotype 020 was examined on the array, the possibility that these 2 PCR-ribotypes may be distinguishable by the presence or absence of the extra 027 genes needs to be further examined. Our study further emphasised that the extra copy of the Agr system (*agrCABD*) present in R20291 [32], and absent in CD630, is present in the majority of strains examined. It is, therefore, most likely not associated with hypervirulence.

Another aim of this study was to determine sequences that could be used to identify the strain CD630. The pCD630 plasmid is only present in one other strain (EK29). As detailed in Table 5, 81 CD630 genes are absent, or highly divergent, from all other PCR-ribotypes (except the PCR-ribotype 012 reference strain). Only the mobile elements CTn*5 *and CTn*7 *do not have any CDS on this list. The only genes which are not derived from mobile elements on this list are CD0211-2, which encode a CTP:phosphocholine cytidylyltransferase and a putative choline sulfatase, and CD2001, CD2003-5, encoding 2 conserved hypothetical proteins, an efflux pump and a MarR transcriptional regulator. CD3136-8 and 3147-53 are included in this list as they are only present in 9 of the 94 strains tested.

**Table 5:** CD630 genes found to be absent from all strains (except CD630 and ECDC 012)

Gene	Synonym	Function
CD0211-2	*licC*	CTP:phosphocholine cytidylyltransferase; putative choline sulfatase

CD0359 - 368		conjugative transposon conserved hypothetical protein; putative transcriptional regulator; two-component system; sensor histidine kinase; two-component system; response regulator; putative lantibiotic ABC transporter; membrane protein; putative lantibiotic ABC transporter;ATP-binding protein; putative lantibiotic ABC transporter; permease protein; putative lantibiotic ABC transporter;ATP-binding protein; two-component system; sensor histidine kinase; two-component system; response regulator

CD0370		putative transcriptional regulator

CD0381-2		putative conjugative transposon replication initiation factor; putative membrane protein

CD0385		putative membrane protein_conjugative transposon protein

CD0495		putative regulatory protein (pseudogene)

CD0498-9	*orf21; orf20*	conjugative transposon FtsK/SpoIIIE-family protein; putative conjugative transposon replication initiation factor

CD0503-4	*orf15; orf14*	conjugative transposon membrane protein; putative cell wall hydrolase

CD0906-907A		putative phage DNA-binding protein; putative phage regulatory protein; putative phage regulatory protein

CD0921		hypothetical phage protein

CD0925-6		hypothetical phage protein; hypothetical phage protein

CD0930-32		phage protein; hyypothetical phage protein; hyypothetical phage protein; hyypothetical phage protein

CD0935		phage modification methylase

CD0958-9		putative oxidoreductase (pseudogene); putative oxidoreductase (pseudogene)

CD0967		hypothetical phage protein

CD0970-1		putative oxidoreductase (pseudogene); putative oxidoreductase (pseudogene)

CD0971		putative oxidoreductase (pseudogene)

CD0975-7		putative oxidoreductase (pseudogene); putative oxidoreductase (pseudogene); putative oxidoreductase (pseudogene)

CD1092A		two-component sensor histidine kinase (pseudogene)

CD1094-99		two-component sensor histidine kinase (pseudogene); putative lantibiotic ABC transporter; permease protein; two-component sensor histidine kinase (pseudogene); two-component sensor histidine kinase (pseudogene); two-component sensor histidine kinase (pseudogene)

CD1102-6		two-component sensor histidine kinase (pseudogene); two-component sensor histidine kinase (pseudogene); two-component sensor histidine kinase (pseudogene); two-component sensor histidine kinase (pseudogene); two-component sensor histidine kinase (pseudogene)

CD1110		two-component sensor histidine kinase (pseudogene)

CD1112		two-component sensor histidine kinase (pseudogene)

CD1115-8		two-component sensor histidine kinase (pseudogene); two-component sensor histidine kinase (pseudogene); two-component sensor histidine kinase (pseudogene); two-component sensor histidine kinase (pseudogene)

CD2001		conserved hypothetical protein

CD2003-5	*effD; effR*	putative efflux pump; MarR-family transcriptional regulator; conserved hypothetical protein

CD2793	*slpA*	cell surface protein (S-layer precursor protein)

CD2890-3		putative oxidoreductase (pseudogene); putative oxidoreductase (pseudogene); putative oxidoreductase (pseudogene); putative oxidoreductase (pseudogene)

CD2897		putative oxidoreductase (pseudogene)

CD2905-7		putative oxidoreductase (pseudogene); putative oxidoreductase (pseudogene); putative oxidoreductase (pseudogene)

CD3136-8	*bglA3*	6-phospho-beta-glucosidase; PTS system; IIabc component; transcription antiterminator

CD3147-53		putative DNA-methyltransferase; hypothetical protein; putative DNA helicase; conserved hypothetical protein; putative phage protein; conserved hypothetical protein; putative phage transcriptional regulator; putative phage DNA-binding protein

CD3326		Integrase

CD3331-3		hypothetical protein; TetR-family transcriptional regulator

CD3341-2		conjugative transposon conserved hypothetical protein (pseudogene); putative phage membrane protein

CD3344		conjugative transposon-related FtsK/SpoIII-relatd protein

CD3347-8		putative membrane protein; conserved hypothetical protein

## Conclusions

*C. difficile *has become the most common cause of nosocomial diarrhoea in recent years, partly due to the emergence and spread of the hypervirulent PCR-ribotype 027. The increasing rates of CDI are not only caused by the spread of this PCR-ribotype, which remains the second most commonly isolated PCR-ribotype in the UK and the fourth most commonly isolated PCR-ribotype in Europe [22,24].

This array comparative genomic study presents a snapshot of current EU clinical strains and the current molecular epidemiology of *C. difficile *[47]. Our study has shown that the PCR-ribotype 027 markers absent in the CD630 genome are not solely confined to PCR-ribotype 027 strains, but appear distributed amongst other PCR-ribotypes to varying degrees. Indeed, in some cases (PCR-ribotype 001, 020 and 106) there is greater overall carriage of these markers (100%) than amongst the PCR-ribotype 027 strains examined (98.8%). The apparent lower carriage rate in the latter may in part be a reflection of the larger sample size analysed (29 × 027) compared to the other PCR-ribotypes (10 × 001, 17 × 106, 9 × 078 and 1 × 020). This does not rule out the possibility that some of these markers may be indicative of increased virulence. Thus, PCR-ribotype 001 is one of the commonest types in Europe, and frequently associated with outbreaks, PCR-ribotype 106 was until recently the epidemic strain in England and Wales [22], whilst PCR-ribotype 078 strains are increasing recognised as being as equally aggressive as PCR-ribotype 027 strains [25]. The presence of markers of enhanced virulence common to 027 would, is, therefore, not surprising.

Although comprehensive and of high density, the microarray employed here is of limited utility value as it does not cover all the extra PCR-ribotype 027 genes later revealed by Stabler *et al*. [32]. The presence of such '027-specific'genes in the PCR-ribotype 078, 001, 020 and 106 should be confirmed. However, as they largely represent transposon-related genes, their usefulness as markers of hypervirulence for diagnostics may be limited.

We have fulfilled the aims of this study by identifying markers for CD630 and markers for hypervirluence, albeit genes that are not just indicative of PCR-ribotype 027. As a consequence of our comprehensive array coverage, we have also defined a more stringent core gene set compared to those previously published [30,32]. Further to this, we have defined a list of genes absent from non-toxinogenic strains and defined the deletion in strain CD37.

## Methods

### Strains and growth conditions

Ninety-four clinical strains were investigated in this study and these included 29 PCR-ribotype 027 strains, 17 PCR-ribotype 106 strains, 10 PCR-ribotype 001 strains, 9 PCR-ribotype 078 strains, 8 PCR-ribotype 002 strains, 8 PCR-ribotype 017 strains and 7 PCR-ribotype 014 strains (Additional file 16). Four non-toxigenic strains were also hybridised to the array for further investigation. The majority of the strains examined this study were isolated in the UK or the Netherlands.

A 10 μl loop was used to inoculate pre-reduced BHIS agar from frozen bacterial stock. The plates were then incubated anaerobically at 37°C under an atmosphere of N_2_:H_2_:CO_2 _(80:10:10, vol:vol:vol) in an anaerobic workstation (Don Whitley, Yorkshire, UK). A single colony was then used to inoculate a 10 ml BHIS broth and incubated overnight prior to DNA extraction.

### DNA Extraction

A traditional DNA extraction method utilising phenol chloroform extraction was used [48]. Briefly, overnight cultures were pelleted and the cells were resuspended in 260 μl buffer EB (Qiagen). After the addition of 20 mg/ml lysozyme (Sigma-Aldrich, Gillingham, Dorset, UK) and 10% SDS (Sigma-Aldrich, U.K.), the solution was incubated at 37°C for 1 hour. The solution was then incubated for a further hour with 100 mg/ml DNase free RNase (Roche, Burgess Hill, U.K.) and Proteinase K (20 mg/ml; Qiagen, Crawley, West Sussex, U.K.). DNA was extracted by phenol:chloroform:IAA (Sigma-Aldrich) washes and phase-lock gel (5 Prime, Gaithersburg, MD, USA). The genomic DNA was then precipitated using ice-cold 100% ethanol and sodium acetate and purified with two washes of 70% ethanol. Purity and quantity were assessed using a NanoDrop1000 spectrophotometer (Thermofisher Scientific, Waltham, MA, USA) and visualisation by gel electrophoresis. Genomic DNA used for hybridisation to the microarray was fragmented by sonication and the fragment size was examined by gel electrophoresis.

### Array design

The array was designed to cover the previously sequenced strain *C. difficile *630, the preliminary 454 sequence data of the 027 strain R20291 and the unannotated sequence of the Canadian 027 isolate QCD32g58 in a strategy similar to that used by Witney *et al *[49]. The R20291 genome sequence was generated by 454/Roche GS20 as discussed in Stabler *et al *[32]. Genome annotation of strain R20291 and QCD32g58 was based on previously published annotations of *C. difficile *strain 630 [17]. The genomic sequences were compared against the database of strain 630 proteins by blastx, and a CDS feature in the query genome was created when a hit of over 90% identity was found. Glimmer3 was used to predict CDSs in genomic regions where no significant hits were found [50]. Any unique genomic regions left were examined and annotated manually in Artemis [51]. The genome comparisons were visualized in Artemis and ACT (Artemis Comparison Tool; [52]. *In silico c*omparison against the Canadian strain QCD32g58 was also performed.

Probes were firstly designed to the CD630 genome and then additional genes of interest from other strains (R20291 and Quebec) were included. The CD630 portion of the array had a tiling design. For this, the genome was divided into 5 Kb segments with the aim of producing the best probe for each 100 bp of sequence. All possible 60 mers were considered and ranked on the basis of melting temperature, likelihood of secondary structure and GC content. The highest ranking probe per 100 bp was then selected. Additional genes were compared to other sequences and any regions of homology discounted. Where possible the 3 highest ranking probes were then selected. Sometimes 3 probes per gene were not possible due to gene length or homology. The array design was completed using the Agilent eArray interactive website. http://www.genomics.agilent.com/CollectionSubpage.aspx?PageType=Product&SubPageType=ProductDetail&PageID=1455

Using this method 40, 0097 oligonucleotides were designed to cover the CD630 genome. A further 681 probes covered any extra genes found in R20291 or QCD32g58. Regions such as the PaLoc were also represented by 346 extra probes at higher density.

### Array production

Our high density custom microarrays were printed using an *in situ *inkjet oligonucleotide synthesizer by Agilent Technologies (Stockport, Chesire [53]. The probes were 60 oligonucleotides in length and printed in single copy per array. Four arrays were printed per slide.

### Labelling and hybridisations

The genomic DNA was labelled using the Bioprime DNA labelling system (Invitrogen, UK). Hybridizations were performed, using SureHyb technology (Agilent, Stockport, Chesire, U.K.), with 2 μg of test genomic DNA labelled with Cy5-dCTP and 2 μg Cy3-dCTP (GE Healthcare Life Sciences, UK) with labelled *C. difficile *630 genomic DNA as a common reference. The labelled DNA was purified using a MiniElute kit (Qiagen, Crawley, W. Sussex, UK) and the extent of Cy dye incorporation was measured using a nanodrop spectrophotometer. The test and control DNA were combined in a final volume of 39 μl and at a concentration of 2 μg each. To this mixture 10× Oligo aCGH/ChIP-on-Chip Blocking agent and 2× Hi-RPM hybridisation buffer (Agilent Technologies, U.K.) were added. The solutions were then denatured at 95°C, and incubated at 37°C for 30 min. The microarray was hybridized overnight using a SureHyb chamber at 65°C for 24 h. Slides were washed once in pre-heated Oligo aCGH/ChIP-on-chip Wash Buffer 1 for 5 min and briefly in Oligo aCGH/ChIP-on-chip Wash Buffer 2. Microarrays were scanned using an Axon 4000b array scanner (Molecular Devices, Sunnyvale, CA, USA) and intensity fluorescence data acquired using GenePix Pro (Molecular Devices).

Technical replicates were performed with our control strains CD630 (self-self hybridisation), R20291 and CD196, and this included dye-swap experiments. No replicates were performed for the clinical strains tested.

### Microarray data analysis

The data was normalized and analysed using GeneSpring GX version 7.3 (Agilent Technologies, UK). Initially for each spot, the median pixel intensity for the local background was subtracted from the median pixel intensity of the spot, and any values less than 0.01 were adjusted to 0.01. Background-subtracted pixel intensities for the test strain channel were divided by those for the reference strain channel. The resulting log ratios were normalised by applying Per Spot Per Chip normalization, using 50% of data from that chip as the median.

An arbitrary cut-off of twofold was used to identify those genes that are specific to one of the strains. Therefore, for all strains, the upper cut-off was set at a ratio of 2 and the lower cut-off at a ratio of 0.5. Genes with a ratio greater than the upper cut-off were deemed to be specific to the test strain, genes with a ratio less than the lower cut-off were deemed to be specific to the reference strain, and genes with ratios between 0.5 and 2 were deemed to be present in both strains. Previous studies have shown that using arbitrary twofold cut-offs to determine presence or absence of genes is more conservative than other methods such as GACK or standard deviation from the median [48]. The presence or absence of a sequence was based on the presence or absence of one probe. The presence of absence of a gene was based on the presence or absence of more than one probe.

### PCR amplification

PCR amplifications were performed using primers described in Supplementary Table 7 and KOD Hot start DNA polymerase (Novagen, Merck Chemicals, UK). Reactions were performed using a denaturation step at 95°C followed by 30 cycles at 95°C for 30 seconds, 52°C for 1 minute, 72°C for 2 - 7 minutes, followed by a final extension of 72°C for 5- 7 minutes. PCRs used to define the PaLoc used the primers and reaction conditions as described by Braun *et al *[45]. PCR primer walking used to confirm the results for the second *agr *locus were performed using the same polymerase as above, with annealing temperatures of 55°C. PCR products were analysed on 1% or 3% agarose gels run at 100-150 mV for 1 hour and stained with ethidium bromide.

### Microarray data accession number

Fully annotated microarray data has been deposited in ArrayExpress (E-MTAB-162).

## Authors' contributions

GLM carried out the majority of the hybridisations, data analysis, data submission and drafted the manuscript. MS and IJD performed *in silico *analysis of R20291 and QCD-32g58 preliminary sequences. IJD and VJW performed initial validation of the microarray. EK provided strains. EK and NPM conceived of the study, and participated in its design and helped to draft the manuscript. All authors read and approved the final manuscript.

## Supplementary Material

Additional file 1 **Lists of probes present and absent for strains CD630, R12087, R20291, R23052, ECDC 001-3, ECDC 012, ECDC 015, ECDC 017 and ECDC 020**. Excel sheets containing presence and absence lists by probe for the above strains. Click here for file

Additional file 2 **Lists of probes present and absent for strains:L22 and L24-33**. Excel sheets containing presence and absence lists by probe for the above strains. Click here for file

Additional file 3 **Lists of probes present and absent for strains EK23-32, EK34 & 35**. Excel sheets containing presence and absence lists by probe for the above strains. Click here for file

Additional file 4 **Lists of probes present and absent for strains L01-11**. Excel sheets containing presence and absence lists by probe for the above strains. Click here for file

Additional file 5 **Lists of probes present and absent for strains L12-21, EK36 and EK37**. Excel sheets containing presence and absence lists by probe for the above strains. Click here for file

Additional file 6 **Lists of probes present and absent for strains 1351(ATCC 43593), CD37, R8366, R20298, R10459, P62, MTZ^R^, L35, L34, L23 and L01**. Excel sheets containing presence and absence lists by probe for the above strains. Click here for file

Additional file 7 **Lists of probes present and absent for strains3232 (ATCC BAA-1801), 7322 (ATCC 43501), DH1396, DH1834, DH482, R23970, R24988, R10432, R108095, R12801 and R15437**. Excel sheets containing presence and absence lists by probe for the above strains. Click here for file

Additional file 8 **Lists of probes present and absent for strains R22079, R24392, R27384 and EK38**. Excel sheets containing presence and absence lists by probe for the above strains. Click here for file

Additional file 9 **List of core genes conserved in all strains by function**. Excel sheets detailing the core genes (by individual probe) conserved by function. Click here for file

Additional file 10 **Table summarising the data for CD630 mobile elements presence and absence by PCR-ribotype**. Summary of the data for CD630 mobile elements presence and absence by PCR-ribotype. Click here for file

Additional file 11 **Table summarising the results for the second *agr *locus in divergent strains**. Details the presence, absence and divergence of each oligonucleotide designed to the second *agr *locus in the strains which show this region to be divergent. Click here for file

Additional file 12 **Table of primers used to confirm PaLoc deletion in the non-toxigenic strains and in the analysis of the second *agr *locus**. Table detailing the primers used to confirm the deletion in the PaLoc of non -toxigenic strains and used in primer walking analysis of the divergent second *agr *locus. Click here for file

Additional file 13 **Probes absent from non-toxigenic strains**. Table detailing the probes absent from the non-toxigenic strains. Click here for file

Additional file 14 **027 genes not represented on the array**. A table detailing the 027 genes identified by Stabler *et al *(56) not represented on the array. Click here for file

Additional file 15 **Ribotype 027 unique genes absent grouped by ribotype**. This file details the 027 unique genes (by probe) identified by Stabler *et al *absent in each PCR-ribotype Click here for file

Additional file 16 **Strains used in this study**. A table listing the strains used in this study, their source and any other information available. Click here for file
