# Supplementary material for: Array comparative hybridisation reveals a high degree of similarity between UK and European clinical isolates of hypervirulent Clostridium difficile
Source: BMC Genomics. 2010 Jun 21;11:389. doi: 10.1186/1471-2164-11-389 (PMC3224701; doi:10.1186/1471-2164-11-389)
Supplement: Additional file 10 — Table summarising the data for CD630 mobile elements presence and absence by PCR-ribotype. Summary of the data for CD630 mobile elements presence and absence by PCR-ribotype. [file 1471-2164-11-389-S10.DOC]

| **Mobile Element** | **Region** | **CD630/ ECDC012** | **027** | **078** | **106** |
| --- | --- | --- | --- | --- | --- |
| CTn1 | CD0354a-86 | + | 25/28 strains negative; CD0371-86 present in strains L14, L20 & R23970 | absent | 8/16 strains negative; CD0371- 85 mixed hybrisation in rest of genes. |
| CTn2 | CD0403-37 | + | absent | absent | absent |
| CTn3 | CD0495-512 | + | absent | only CD0496-7, CD0504-11 absent, mixed hybridised in rest of genes of genes | absent |
| Prophage 1 | CD0904-979 | + | absent | absent | absent |
| CTn4 | CD1090-1118 | + | Absent; L04 majority positive hybridisation | absent | absent |
| CTn5 | CD1845-78a | + | absent from 2 strains; CD1864-69 only absent from rest | 2/8 strains CD1849-63 only absent; rest negative | All absent except L25 where only 1864a-77 absent |
| Prophage 2 | CD2889-2952 | + | absent | limited hybridisation in 2/9 strains between CD2927-52 | absent |
| CTn6 | CD3325-49 | + | absent | absent | absent |
| CTn7 | CD3369-93 | + | 3 strains divergent CD3374-92, rest absent | 2 strains absent; CD3361-72, CD3378-82 & CD3383-92 absent in rest | 2 strains absent; CD3361-73, CD3378-82 & CD3383-92 absent in rest |

| **Mobile Element** | **Region** | **CD630/ ECDC012** | **001** | **002** | **NT strains** |
| --- | --- | --- | --- | --- | --- |
| CTn1 | CD0354a-86 | + | 8/10 strains negative; CD0371-85 mixed hybrisation in rest of genes. | absent | absent in 2/4 strains; CD0354/5 and CD0371-86 mixed hybrisation in rest of genes. |
| CTn2 | CD0403-37 | + | absent | absent | absent |
| CTn3 | CD0495-512 | + | absent | absent | absent; 1351 only CD0496-7, CD0504a-11 absent, mixed hybridised in rest of genes |
| Prophage 1 | CD0904-979 | + | absent | absent | absent |
| CTn4 | CD1090-1118 | + | Absent; L27 majority positive hybridisation | absent | present in 7322 only |
| CTn5 | CD1845-78a | + | absent | absent | absent |
| Prophage 2 | CD2889-2952 | + | limited hybridisation in 2/10 strains between CD2979-52 | limited hybridisation in 2/8 strains between CD2927-52 | limited hybridisation in 1/4 strains between CD2979-52 |
| CTn6 | CD3325-49 | + | absent | absent | absent |
| CTn7 | CD3369-93 | + | 1 strain absent; 4 strains CD3378-81, CD3383-91 absent, rest present | absent | absent 2/3 |

| **Mobile Element** | **Region** | **CD630/ ECDC012** | **014** | **015*** | **017** |
| --- | --- | --- | --- | --- | --- |
| CTn1 | CD0354a-86 | + | absent 2/7 stains; CD0354/5 and CD0371-86 mixed hybrisation in rest of genes. | negative | 2/6 strains negative; CD0354/5 and CD0371-86 mixed hybrisation in rest of strains. |
| CTn2 | CD0403-37 | + | absent | absent | absent |
| CTn3 | CD0495-512 | + | absent | absent | absent |
| Prophage 1 | CD0904-979 | + | absent | absent | absent |
| CTn4 | CD1090-1118 | + | absent | absent | absent |
| CTn5 | CD1845-78a | + | absent | absent | Only CD1864-9 absent in all strains |
| Prophage 2 | CD2889-2952 | + | limited hybridisation in 5/7 strains between CD2979-52 | limited hybridisation between CD2927-52 | absent |
| CTn6 | CD3325-49 | + | only EK15 shows mixed hybridisation between CD3330-44; rest absent | absent | absent |
| CTn7 | CD3369-93 | + | absent 2 strains, present other strains | absent | 2 strains all absent, CD3373-92 genes divergent |

| **Mobile Element** | **Region** | **CD630/ ECDC012** | **020*** | **003*** | **Notes** |
| --- | --- | --- | --- | --- | --- |
| CTn1 | CD0354a-86 | + | mixed hybrisation in CD0354/5 and CD0371a- 86 | mixed hybrisation in CD0354/5 and CD0371absent 86 | Mixed hybrisation: negative and 1:1 in most strains |
| CTn2 | CD0403-37 | + | absent | absent | 4/9 CD0406 probes present in all strains |
| CTn3 | CD0495-512 | + | absent | absent |  |
| Prophage 1 | CD0904-979 | + | absent | absent |  |
| CTn4 | CD1090-1118 | + | absent | absent | 11/14 CD1090 probes present in all strains |
| CTn5 | CD1845-78a | + | absent | absent |  |
| Prophage 2 | CD2889-2952 | + | limited hybridisation between CD2979-52 | absent |  |
| CTn6 | CD3325-49 | + | absent | absent | all strains hybridise to partial CD3325 & CD3349 |
| CTn7 | CD3369-93 | + | present | absent | CD3369 & CD3393 present all |

Absent means absent or highly divergent

* Only one strain of these ribotypes tested.

+ present
