# Supplementary material for: Array comparative hybridisation reveals a high degree of similarity between UK and European clinical isolates of hypervirulent Clostridium difficile
Source: BMC Genomics. 2010 Jun 21;11:389. doi: 10.1186/1471-2164-11-389 (PMC3224701; doi:10.1186/1471-2164-11-389)
Supplement: Additional file 11 — Table summarising the results for the second agr locus in divergent strains. Details the presence, absence and divergence of each oligonucleotide designed to the second agr locus in the strains which show this region to be divergent. [file 1471-2164-11-389-S11.DOC]

| P= signal over 2 fold |
| --- |
| D= signal around 1:1 |
| N= signal less than 0.5 |
