# Supplementary material for: Array comparative hybridisation reveals a high degree of similarity between UK and European clinical isolates of hypervirulent Clostridium difficile
Source: BMC Genomics. 2010 Jun 21;11:389. doi: 10.1186/1471-2164-11-389 (PMC3224701; doi:10.1186/1471-2164-11-389)
Supplement: Additional file 12 — Table of primers used to confirm PaLoc deletion in the non-toxigenic strains and in the analysis of the second agr locus. Table detailing the primers used to confirm the deletion in the PaLoc of non -toxigenic strains and used in primer walking analysis of the divergent second agr locus. [file 1471-2164-11-389-S12.DOC]

| Primer name | Sequence | Target |
| --- | --- | --- |
| Tim 5 | CCACAGATGCTTTTAGCAGGAA | PaLoc |
| Struppi5 | tccaatcactgctccagctat | PaLoc |
| lok3 | tttaccagaaaaagtagctttaa | PaLoc |
| tim3 | AAAAGCGATGCTATTATAGTCAAA | PaLoc |
| STRUPPI3 | CCTTATTAACAGCTTGTCTAGAT | PaLoc |
| TIM0 | CAAGATGATTTAGTGATATCAGAA | PaLoc |
| STRUPPI0 | CATAAGTTCCTCCTGAACCATA | PaLoc |
| TIM 1 | GTTTAAGTGCAATAAAAAGTCGTA | PaLoc |
| STRUPPI1 | GGTAATCCACATAAGCACATATT | PaLoc |
| TIM4 | taagctaaaagcaaagtccaaga | PaLoc |
| struppi4 | TTATTTCTTTTGTAATATGTTCGCT | PaLoc |
| tim2 | GCACCTCATCACCATCTTCAA | PaLoc |
| STRUPPI2 | TGAAGACCATGAGGAGGTCAT | PaLoc |
| LOK2 | TTAGAGCTTCCTTTTCCTTCATT | PaLoc |
| LOK1 | AAAATATACTGCACATCTGTATAC | PaLoc |
| TIM6 | TCCAATATAATAAATTAGCATTCCA | PaLoc |
| STRUPPI6 | GGCTATTACACGTAATCCAGATA | PaLoc |
| CR3184F | CATTAGTGGAAATGTTGTATACCATAATAATGTAG | CDR3184 |
| CDR3185R | GTATAGCACAGGCTTTAATAGGTGATTCT | CDR3185 |
| CDR3186R | CGAGGTGACATTAGATATAGAGTATGAAGG | CDR3186 |
| CDR3187R | GTTATTGCACTTTAAGACAGTTCACAG | CDR3187 |
| CDR3187AR | CAGTGCAAGTTCTTGGGTAGCACATC | CDR3187A |
| CDR3188R | GGCTAGGATTAATGATTGCTGAAGGTTCTG | CDR3188 |
| CDR3189R | TAAATGGTATGGATACTGCTAGGAAGATAAGAG | CDR3189 |
| CDR3140F | TACCTTCCTAGATAAACCTAGTTTCTC | CDR3140 |
| CDR3140R | ATACCTAATTACAGGTAACTGGGAGAT | CDR3140 |
