# Supplementary material for: Array comparative hybridisation reveals a high degree of similarity between UK and European clinical isolates of hypervirulent Clostridium difficile
Source: BMC Genomics. 2010 Jun 21;11:389. doi: 10.1186/1471-2164-11-389 (PMC3224701; doi:10.1186/1471-2164-11-389)
Supplement: Additional file 14 — 027 genes not represented on the array. A table detailing the 027 genes identified by Stabler et al (56) not represented on the array. [file 1471-2164-11-389-S14.DOC]

| **R20291** | **CD196** | **Fn Cat** | **Function** |
| --- | --- | --- | --- |
| 1. CDR20291_0224 | CD196_0237 | 3.3.18 | glucose-1-phosphate thymidylyltransferase |
| 1. CDR20291_0377 | CD196_0390 | 5.1.4 | transposase-like protein b |
| 1. CDR20291_0383 | CD196_0397 | 0.0.2 | putative uncharacterized protein |
| 1. CDR20291_0405 | CD196_0419 | 5.1.4 | transposase-like protein b |
| 1. CDR20291_0439 | CD196_0453 | 6.1.1 | sensor histidine kinase |
| 1. CDR20291_0440 | CD196_0454 | 4.1.6 | cell surface protein (putative hemagglutinin/adhesin) |
| 1. CDR20291_0500 | CD196_0516 | 5.1.4 | putative uncharacterized protein |
| 1. CDR20291_0514 | CD196_0530 | 5.1.4 | transposase-like protein b |
| 1. CDR20291_0528 | CD196_0544 | 0.0.2 | putative uncharacterized protein |
| 1. CDR20291_0530 | CD196_0546 | 5.1.4 | transposase-like protein b |
| 1. CDR20291_0569 | CD196_0587 | 7.0.0 | fic family protein; putative filamentation induced by cAMP protein |
| 1. CDR20291_0667 | CD196_0686 | 0.0.0 | [no /product] |
| 1. CDR20291_0697 | CD196_0716 | 5.1.4 | transposase-like protein b |
| 1. CDR20291_0809 | CD196_0829 | 5.1.4 | transposase-like protein b |
| 1. CDR20291_0826 | CD196_0845 | 5.1.4 | 3-isopropylmalate dehydrogenase |
| 1. CDR20291_0959 | CD196_0981 | 5.1.4 | transposase-like protein b |
| 1. CDR20291_0979 | CD196_1001 | 0.0.0 | [no /product] |
| 1. CDR20291_1071 | CD196_1093 | 0.0.2 | putative uncharacterized protein |
| 1. CDR20291_1072 | CD196_1094 | 5.1.4 | integrase, catalytic region |
| 1. CDR20291_1184 | CD196_1206 | 0.0.0 | [no /product] |
| 1. CDR20291_1338 | CD196_1361 | 5.1.4 | transposase-like protein b |
| 1. CDR20291_1431 | CD196_1456 | 5.1.2 | putative phage DNA-binding protein |
| 1. CDR20291_1432 | CD196_1457 | 5.1.2 | phage terminase large subunit |
| 1. CDR20291_1433 | CD196_1458 | 5.1.2 | phage portal protein |
| 1. CDR20291_1434 | CD196_1459 | 5.1.2 | phage protein |
| 1. CDR20291_1435 | CD196_1460 | 5.1.2 | scaffold protein |
| 1. CDR20291_1436 | CD196_1461 | 5.1.2 | putative phage major capsid protein |
| 1. CDR20291_1437 | CD196_1462 | 5.1.2 | hypothetical phage protein |
| 1. CDR20291_1438 | CD196_1463 | 5.1.2 | phage protein |
| 1. CDR20291_1440 | CD196_1465 | 5.1.2 | phage protein |
| 1. CDR20291_1441 | CD196_1466 | 5.1.2 | phage protein |
| 1. CDR20291_1442 | CD196_1467 | 5.1.2 | phage protein |
| 1. CDR20291_1444 | CD196_1469 | 5.1.2 | phage protein |
| 1. CDR20291_1445 | CD196_1470 | 5.1.2 | hypothetical phage protein |
| 1. CDR20291_1450 | CD196_1475 | 5.1.2 | putative phage cell wall hydrolase |
| 1. CDR20291_1451 | CD196_1476 | 5.1.2 | putative phage cell wall hydrolase |
| 1. CDR20291_1453 | CD196_1478 | 5.1.2 | phage protein |
| 1. CDR20291_1454 | CD196_1479 | 5.1.2 | putative phage tail protein |
| 1. CDR20291_1455 | CD196_1480 | 5.1.2 | phage protein |
| 1. CDR20291_1458 | CD196_1483 | 5.1.2 | putative uncharacterized protein |
| 1. CDR20291_1461 | CD196_1486 | 5.1.2 | putative uncharacterized protein |
| 1. CDR20291_1469 | CD196_1494 | 5.1.4 | transposase-like protein b |
| 1. CDR20291_1906 | CD196_1863 | 5.1.4 | transposase-like protein b |
| 1. CDR20291_1923 | CD196_1880 | 6.3.12 | hth transcriptional regulator merR family |
| 1. CDR20291_2196 | CD196_2150 | 5.1.4 | integrase, catalytic region |
| 1. CDR20291_2197 | CD196_2151 | 5.1.4 | transposase |
| 1. CDR20291_2198 | CD196_2152 | 0.0.0 | hypothetical protein |
| 1. CDR20291_2279 | CD196_2231 | 1.4.0 | putative beta-lactamase inducer |
| 1. CDR20291_2280 | CD196_2232 | 1.4.0 | putative beta-lactamase repressor |
| 1. CDR20291_2501 | CD196_2454 | 5.1.4 | transposase-like protein b |
| 1. CDR20291_2674 | CD196_2627 | 4.1.6 | putative membrane protein |
| 1. CDR20291_2716 | CD196_2669 | 5.1.4 | transposase-like protein b |
| 1. CDR20291_2834 | CD196_2786 | 5.1.4 | transposase-like protein b |
| 1. CDR20291_2950 | CD196_2902 | 5.1.4 | transposase-like protein b |
| 1. CDR20291_2993 | CD196_2946 | 0.0.2 | putative uncharacterized protein |
| 1. CDR20291_3276 | CD196_3230 | 5.1.4 | transposase, mutator type |
| 1. CDR20291_3277 | CD196_3231 | 4.1.7 | putative exported protein |
| 1. CDR20291_3279 | CD196_3233 | 6.5.0 | putative regulatory protein |
| 1. CDR20291_3282 | CD196_3236 | 0.0.2 | putative uncharacterized protein |
| 1. CDR20291_3290 | CD196_3244 | 5.1.4 | transposase mutator type |
| 1. CDR20291_3453 | CD196_3407 | 5.1.5 | putative collagen-binding surface protein |
| 1. CDR20291_3457 | CD196_3411 | 0.0.2 | putative uncharacterized protein |
| 1. CDR20291_3460 | CD196_3414 | 5.1.4 | conjugative transposon protein |
| 1. CDR20291_3472 | CD196_3426 | 1.5.0 | putative abc transporter, permease protein |
| 1. CDR20291_3473 | CD196_3427 | 0.0.2 | putative uncharacterized protein |
