# Supplementary material for: Array comparative hybridisation reveals a high degree of similarity between UK and European clinical isolates of hypervirulent Clostridium difficile
Source: BMC Genomics. 2010 Jun 21;11:389. doi: 10.1186/1471-2164-11-389 (PMC3224701; doi:10.1186/1471-2164-11-389)
Supplement: Additional file 16 — Strains used in this study. A table listing the strains used in this study, their source and any other information available. [file 1471-2164-11-389-S16.DOC]

Bacterial strains used in this study

| **Strain** | **Source** | **PCR-ribotype; Notes** |
| --- | --- | --- |
| 1. CD630 | P. Mullany, UCL | 012 |
| 1. R20291 | J. Brazier, ARU^§^ | 027; Toxin A+, toxin B+, cdtA+, cdtB+ |
| 1. L24 | LUMC | 001; Toxin A+, toxin B+, cdtA-, cdtB+ |
| 1. L25 | LUMC | 001; Toxin A+, toxin B+, cdtA-, cdtB+ |
| 1. L26 | LUMC | 001; Toxin A+, toxin B+, cdtA-, cdtB+ |
| 1. L27 | LUMC | 001; Toxin A+, toxin B+, cdtA-, cdtB+ |
| 1. L28 | LUMC | 001; Toxin A+, toxin B+, cdtA-, cdtB+ |
| 1. L29 | LUMC | 001; Toxin A+, toxin B+, cdtA-, cdtB- |
| 1. L30 | LUMC | 001; Toxin A+, toxin B+, cdtA-, cdtB- |
| 1. R8366 | J. Brazier,, ARU^§^ | 001 |
| 1. 001 | EU reference set* | 001 |
| 1. P62 | M. Wilcox, University of Leeds | 001; Toxin A+, toxin B+ |
| 1. EK2 | LUMC | 002 |
| 1. EK3 | LUMC | 002 |
| 1. EK4 | LUMC | 002 |
| 1. EK5 | LUMC | 002 |
| 1. EK6 | LUMC | 002 |
| 1. EK7 | LUMC | 002 |
| 1. EK8 | LUMC | 002 |
| 1. ECDC 002 | EU reference set* | 002 |
| 1. EK9 | LUMC | 014 |
| 1. EK14 | LUMC | 014 |
| 1. EK15 | LUMC | 014 |
| 1. EK16 | LUMC | 014 |
| 1. L31 | LUMC | 014; Toxin A+, toxin B+, cdtA-, cdtB- |
| 1. L32 | LUMC | 014; Toxin A+, toxin B+, cdtA-, cdtB- |
| 1. L33 | LUMC | 014; Toxin A+, toxin B-, cdtA-, cdtB- |
| 1. L22 | LUMC | 017; Toxin A-, toxin B+, cdtA-, cdtB- |
| 1. L23 | LUMC | 017; Toxin A-, toxin B+, cdtA-, cdtB- |
| 1. 017 | EU reference set* | 017; |
| 1. EK19 | LUMC | 017 |
| 1. EK20 | LUMC | 017 |
| 1. L34 | LUMC | 078; Toxin A+, toxin B+, cdtA+, cdtB+ |
| 1. L35 | LUMC | 078; Toxin A+, toxin B+, cdtA+, cdtB+ |
| 1. 078 | EU reference set* | 078 |
| 1. EK23 | LUMC | 078 |
| 1. EK24 | LUMC | 078 |
| 1. EK26 | LUMC | 078 |
| 1. EK27 | LUMC | 078 |
| 1. EK28 | LUMC | 078 |
| 1. EK29 | LUMC | 078 |
| 1. EK30 | LUMC | 106 |
| 1. EK31 | LUMC | 106 |
| 1. EK32 | LUMC | 106 |
| 1. EK34 | LUMC | 106 |
| 1. EK35 | LUMC | 106 |
| 1. EK36 | LUMC | 106 |
| 1. EK37 | LUMC | 106 |
| 1. EK38 | LUMC | 106 |
| 1. R10432 | V. Hall, ARU^§^ | 106 |
| 1. R12801 | V. Hall, ARU^§^ | 106 |
| 1. R15347 | V. Hall, ARU^§^ | 106 |
| 1. R108095 | V. Hall, ARU^§^ | 106 |
| 1. R22079 | V. Hall, ARU^§^ | 106 |
| 1. R23942 | V. Hall, ARU^§^ | 106 |
| 1. R27384 | V. Hall, ARU^§^ | 106 |
| 1. R10459 | V. Hall, ARU^§^ | 106 |
| 1. L1 | LUMC | 027; Toxin A+, toxin B+, cdtA+, cdtB+ |
| 1. L2 | LUMC | 027; Toxin A+, toxin B+, cdtA+, cdtB+ |
| 1. L3 | LUMC | 027; Toxin A+, toxin B+, cdtA+, cdtB+ |
| 1. L4 | LUMC | 027; Toxin A+, toxin B+, cdtA+, cdtB+ |
| 1. L5 | LUMC | 027; Toxin A+, toxin B+, cdtA+, cdtB+ |
| 1. L6 | LUMC | 027; Toxin A+, toxin B+, cdtA+, cdtB+ |
| 1. L7 | LUMC | 027; Toxin A+, toxin B+, cdtA+, cdtB+ |
| 1. L8 | LUMC | 027; Toxin A+, toxin B+, cdtA+, cdtB+ |
| 1. L9 | LUMC | 027; Toxin A+, toxin B+, cdtA+, cdtB+ |
| 1. L10 | LUMC | 027; Toxin A+, toxin B+, cdtA+, cdtB+ |
| 1. L11 | LUMC | 027; Toxin A+, toxin B+, cdtA+, cdtB+ |
| 1. L12 | LUMC | 027; Toxin A+, toxin B+, cdtA+, cdtB+ |
| 1. L13 | LUMC | 027; Toxin A+, toxin B+, cdtA+, cdtB+ |
| 1. L14 | LUMC | 027; Toxin A+, toxin B+, cdtA+, cdtB+ |
| 1. L15 | LUMC | 027; Toxin A+, toxin B+, cdtA+, cdtB+ |
| 1. L16 | LUMC | 027; Toxin A+, toxin B+, cdtA+, cdtB+ |
| 1. L17 | LUMC | 027; Toxin A+, toxin B+, cdtA+, cdtB+ |
| 1. L19 | LUMC | 027; Toxin A+, toxin B+, cdtA+, cdtB+ |
| 1. L20 | LUMC | 027: CDC 32 (Historical US strain) ; Toxin A+, toxin B+, cdtA+, cdtB+ |
| 1. L21 | LUMC | 027: CDC 38 (New US strain) ;  Toxin A+, toxin B+, cdtA+, cdtB+ |
| 1. DH482 | V. Hall, ARU^§^ | 027 |
| 1. DH1396 | V. Hall, ARU^§^ | 027 |
| 1. R24988 | V. Hall, ARU^§^ | 027 |
| 1. R23970 | V. Hall, ARU^§^ | 027 |
| 1. R20352 | J. Brazier,, ARU^§^ | 027; Canadian |
| 1. R20298 | J. Brazier, ARU^§^ | 027; USA |
| 1. R12087 | J. Brazier, ARU^§^ | 027; Historical Popoff isolate, CD196 (40) |
| 1. CD37 | P. Mullany,UCL | Toxin negative |
| 1. Mtz^R^ | P. Mastrantonio^¶^ |  |
| 1. ATCC 43601 | Braun *et al*., 1996 | Toxin negative (1351) |
| 1. ATCC 43593 | Braun *et al*., 1996 | Toxin negative (3232) |
| 1. ATCC BAA-1801 | Braun *et al*., 1996 | Toxin negative (3722) |
| 1. 015 | EU reference set* | 015 |
| 1. 020 | EU reference set* | 020 |
| 1. 003 | EU reference set* | 003 |
| 1. 012 | EU reference set* | 012 |
| 1. 014 | EU reference set* | 014 |

*(5)

^§^Anaerobe Reference Unit, Department of Medical Microbiology and Public Health Laboratory, University Hospital of Wales, Cardiff CF4 4XW, United Kingdom

^¶^ Istituto Superiore di Sanità, Viale Regina Elena 299 00161 - Roma (I)
